# Interaction effects of significant risk factors on overweight or obesity among 7222 preschool–aged children from Beijing

**DOI:** 10.18632/aging.103701

**Published:** 2020-08-03

**Authors:** Bo Zhou, Yuan Yuan, Kundi Wang, Wenquan Niu, Zhixin Zhang

**Affiliations:** 1 Graduate School, Beijing University of Chinese Medicine, Beijing, China; 2 International Medical Services, China–Japan Friendship Hospital, Beijing, China; 3 Department of Pediatrics, China–Japan Friendship Hospital, Beijing, China; 4 Institute of Clinical Medical Sciences, China–Japan Friendship Hospital, Beijing, China

**Keywords:** childhood overweight or obesity, preschool–aged children, risk, interaction, nomogram model

## Abstract

Objectives: We aimed to identify potential risk factors, both individually and interactively, associated with overweight and obesity among preschool–aged children, and further to create a risk prediction nomogram model.

Results: After graded multivariable adjustment, maternal body mass index (BMI) (odds ratio, 95% confidence interval, P under China criteria: 1.07, 1.05 to 1.10, <0.001), maternal pre–pregnancy BMI (1.08, 1.05 to 1.10, <0.001), breastfeeding duration (0.86, 0.76 to 0.98, 0.019), and sleep duration (0.95, 0.90 to 1.00, 0.042) were found to be independently and consistently associated with the significant risk of childhood overweight or obesity under three different growth criteria. Further analyses revealed the four significant factors acted in an additive manner, especially for the interaction between maternal obesity, sleep duration, and breastfeeding. Finally, a risk prediction nomogram model was created for childhood overweight or obesity based on significant and conventional attributes under each criterion.

Conclusions: Our findings provide evidence that the four significant factors are associated with the risk of childhood overweight or obesity in an additive manner.

Methods: Using a stratified cluster random sampling strategy, 7222 preschool–aged children were analyzed. Childhood overweight and obesity are defined according to the China criteria and two widely–used international growth criteria.

## INTRODUCTION

Concern is growing over the increasing prevalence of childhood overweight or obesity worldwide during the past few decades [1, 2]. The Global Burden of Disease Study 2013 estimated the global prevalence of overweight or obesity in children and adolescents from developed countries at 23.8% in boys and 22.6% in girls, and from developing countries at 12.9% in boys and 13.4% in girls [3]. In China, national survey data indicated an increasing prevalence of childhood overweight or obesity from 11.7% to 25.2% during 1991–2011 [4]. In view of the detrimental effects of childhood obesity on the cognitive, behavioral, and social–emotional development in children [5, 6], as well as on the subsequent disease burden of disability and premature death from cardiometabolic diseases, cancer, and musculoskeletal disorders in adulthood [7–10], more attention should be directed towards the epidemiologic risk profiling of childhood overweight or obesity, which might give us an insight into the development of preventive and intervention strategies to curb this global burden.

In current medical literature, considerable interest remains in the identification of potential risk factors attributable to the development of overweight or obesity in children. For example, some epidemiologic studies reported significant associations of higher maternal gestational weight gain with higher weight and consequent obesity risk in children [11, 12]. Additionally, Hills and colleagues have written an excellent review on childhood obesity, underscoring the important role of physical activity in the prevention of overweight and obesity in childhood and adolescence [13]. We recently, in a cross–sectional survey of 1333 Chinese preschool–aged children from 5 kindergartens of Chaoyang District, Beijing, demonstrated that bedtime synergistically interacted with eating speed when predicting childhood overweight and obesity [14]. Despite many research endeavors, we are still facing challenges in predicting children who are more likely to be overweight or obese. The most compelling reason might be due to the complex processes underlying obesity etiology, on which it is unlikely that any one single predictor would have a dramatic impact. Given this complexity, it is of great importance to construct a robust prediction model via incorporating multiple established risk factors for childhood overweight or obesity. However, the available literature on this subject is scarce.

To fill this gap in our knowledge and yield more information for future studies, we conducted a cross–sectional investigation among preschool–aged children who were enrolled from 20 kindergartens in Beijing, aiming to identify potential risk factors, both in isolation and in combination, associated with childhood overweight and obesity. To further enhance the applicability of our findings, we herein prepared to create a risk prediction nomogram model on the basis of promising significant attributes.

## RESULTS

### Baseline characteristics

Supplementary Table 1 shows the baseline characteristics of 7222 study children upon stratification by three different growth criteria. Children with overweight and obesity were combined because of small sample sizes.

### Identification of significant risk factors

As shown in Table 1, eight factors, including maternal education, family income, maternal pre–pregnancy smoking, maternal BMI, maternal pre–pregnancy BMI, breastfeeding duration, GWG, and sleep duration were found to be associated with the significant risk of childhood overweight or obesity before adjustment at a significance level of 5% under three different growth criteria. After adjusting for age of children, sex, and region, statistical significance was retained for all eight factors except maternal pre–pregnancy smoking under all growth criteria. Further additional adjustment for birthweight, delivery mode, maternal age, paternal age, gestational diabetes mellitus, and gestational hypertension showed that only four factors were independently and consistently associated with the significant risk of childhood overweight or obesity under three different growth criteria, including maternal BMI (for example, OR, 95% CI, P under China criteria: 1.07, 1.05 to 1.10, <0.001), maternal pre–pregnancy BMI (1.08, 1.05 to 1.10, <0.001), breastfeeding duration (0.86, 0.76 to 0.98, 0.019), and sleep duration (0.95, 0.90 to 1.00, 0.042).

**Table 1:** Identification of potential risk factors for overweight or obesity in preschool–aged children under three different growth criteria.

**Variables**	**China criteria**			**WHO criteria**			**IOTF criteria**		
	**OR**	**95% CI**	**P**	**OR**	**95% CI**	**P**	**OR**	**95% CI**	**P**
**Unadjusted**									
Maternal education	1.25	1.15 to 1.37	<0.001	1.34	1.20 to 1.49	<0.001	1.33	1.20 to 1.48	<0.001
Family income	1.17	1.10 to 1.25	<0.001	1.20	1.12 to 1.30	<0.001	1.23	1.14 to 1.32	<0.001
Maternal pre–pregnancy smoking	1.46	1.03 to 2.08	0.034	1.52	1.00 to 2.30	0.050	1.61	1.08 to 2.39	0.019
Maternal BMI	1.09	1.07 to 1.11	<0.001	1.09	1.06 to 1.11	<0.001	1.09	1.07 to 1.12	<0.001
Maternal pre–pregnancy BMI	1.09	1.07 to 1.11	<0.001	1.08	1.06 to 1.10	<0.001	1.09	1.07 to 1.12	<0.001
Breastfeeding duration	0.82	0.74 to 0.92	0.001	0.80	0.70 to 0.92	0.002	0.78	0.68 to 0.89	<0.001
Gestational weight gain	1.36	1.20 to 1.54	<0.001	1.33	1.14 to 1.56	<0.001	1.39	1.20 to 1.61	<0.001
Sleep duration	0.92	0.88 to 0.97	0.001	0.86	0.81 to 0.91	<0.001	0.89	0.84 to 0.94	<0.001
**Age–, gender–, and region–adjusted**									
Maternal education	1.19	1.09 to 1.31	<0.001	1.25	1.11 to 1.40	<0.001	1.26	1.13 to 1.40	<0.001
Family income	1.13	1.06 to 1.20	<0.001	1.13	1.04 to 1.23	0.003	1.16	1.08 to 1.26	<0.001
Maternal pre–pregnancy smoking	1.46	1.02 to 2.09	0.036	1.51	0.99 to 2.32	0.058	1.65	1.11 to 2.46	0.014
Maternal BMI	1.09	1.07 to 1.11	<0.001	1.08	1.06 to 1.11	<0.001	1.09	1.07 to 1.11	<0.001
Maternal pre–pregnancy BMI	1.09	1.07 to 1.11	<0.001	1.08	1.06 to 1.11	<0.001	1.09	1.07 to 1.11	<0.001
Breastfeeding duration	0.83	0.74 to 0.93	0.001	0.84	0.73 to 0.96	0.013	0.79	0.69 to 0.90	0.001
Gestational weight gain	1.30	1.15 to 1.48	<0.001	1.24	1.06 to 1.45	0.008	1.32	1.13 to 1.53	<0.001
Sleep duration	0.94	0.90 to 0.98	0.009	0.94	0.88 to 1.00	0.035	0.91	0.86 to 0.97	0.002
**Multivariable adjusted**									
Maternal education	1.07	0.94 to 1.22	0.315	1.20	1.06 to 1.36	0.005	1.02	0.87 to 1.19	0.807
Family income	1.06	0.99 to 1.15	0.114	1.12	1.02 to 1.22	0.013	1.06	0.97 to 1.16	0.212
Maternal pre–pregnancy smoking	1.41	0.94 to 2.10	0.094	1.39	0.87 to 2.22	0.174	1.49	0.95 to 2.34	0.084
Maternal BMI	1.07	1.05 to 1.10	<0.001	1.08	1.06 to 1.11	<0.001	1.07	1.05 to 1.10	<0.001
Maternal pre–pregnancy BMI	1.08	1.05 to 1.10	<0.001	1.08	1.06 to 1.11	<0.001	1.07	1.05 to 1.10	<0.001
Breastfeeding duration	0.86	0.76 to 0.98	0.019	0.83	0.71 to 0.96	0.015	0.80	0.69 to 0.92	0.002
Gestational weight gain	1.14	1.00 to 1.31	0.059	1.13	0.95 to 1.34	0.165	1.14	0.97 to 1.34	0.116
Sleep duration	0.95	0.90 to 1.00	0.042	0.94	0.89 to 0.98	0.048	0.94	0.88 to 0.99	0.029

Abbreviations: OR, odds ratio; 95% CI, 95% confidence interval; BMI, body mass index; WHO, World Health Organization; IOTF, International Obesity Task Force. P values were calculated after adjusting for age of children, sex, region, birthweight, delivery mode, maternal age, paternal age, gestational diabetes mellitus and gestational hypertension.

### Prediction accuracy assessment

To assess the prediction accuracy of the four significant factors identified, two models were constructed, *viz*. the basic model and the full model. The full model included all variables under investigation in this survey, and the basic model included all variables except the four significant factors. Both calibration and discrimination statistics were used to assess the prediction accuracy gained by adding the four significant factors to the basic model under three different growth criteria (Table 2). Prediction accuracy was significantly improved in the full model relative to the basic model. For example, as revealed by the −2 log likelihood ratio test, both models differed significantly in prediction performance under three different growth criteria (all P <0.0001).

**Table 2:** Prediction accuracy gained by adding the four significant factors identified for overweight or obesity in preschool–aged children under three different growth criteria.

**Statistics**	**China criteria**		**WHO criteria**		**IOTF criteria**	
	**Basic model**	**Full model**	**Basic model**	**Full model**	**Basic model**	**Full model**
**Calibration**						
AIC	5236	4994	3665	3515	4062	3876
BIC	5418	5201	3847	3721	4244	4083
LR test (χ^2^)	Ref.	48.18	Ref.	27.55	Ref.	36.14
LR test (P value)	Ref.	<0.0001	Ref.	<0.0001	Ref.	<0.0001
**Discrimination**						
NRI (P value)	Ref.	0.0111	Ref.	0.0093	Ref.	0.0062
IDI (P value)	Ref.	<0.0001	Ref.	0.0006	Ref.	<0.0001
AUROC (P value)	0.0350		0.0382		0.0352	

Abbreviations: AIC, Akaike information criterion; BIC, Bayesian information criterion; LR, likelihood ratio; NRI, net reclassification improvement; IDI, integrated discrimination improvement; AUROC, area under the receiver operating characteristic; Ref., reference; WHO, World Health Organization; IOTF, International Obesity Task Force.

**Figure 1:**
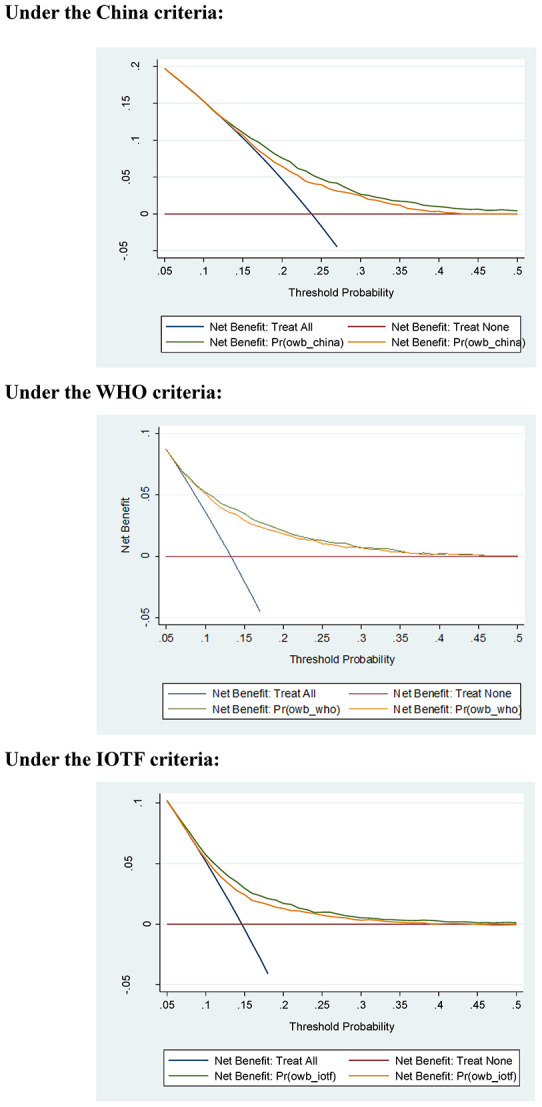
**Net benefits gained by the four significant factors identified for childhood overweight or obesity in decision curve analysis under three different growth criteria.** Abbreviations: WHO, World Health Organization; IOTF, International Obesity Task Force.

Additionally, decision curve analysis indicated that the net benefits gained by adding the four significant factors to the basic model were obvious under three different growth criteria (Figure 1).

### Interaction explorations

Given that childhood overweight or obesity development is a complex process, the effect of any risk factor may be small when assessed individually, and it may be more pronounced in the presence of other risk factors. To yield more information, pair–wise interactions of the four significant factors identified were explored, as shown in Table 3. To increase the power for detecting significant results, the four significant factors were divided into two or three groups. Specifically, both maternal BMI and maternal pre–pregnancy BMI were divided into the low (BMI <18.5 kg/m^2^), normal (BMI: 18.5–24 kg/m^2^), and high (BMI ≥24 kg/m^2^) groups [15]. Breastfeeding duration was binarized on the basis of its mean value (10 months). Sleep duration was binarized on the basis of 10 hours according to the consensus statement of the American Academy of Sleep Medicine, that is, children 3 to 5 years of age should sleep 10 to 13 hours per 24 hours (including naps) on a regular basis to promote optimal health [16].

**Table 3:** The interaction of the four significant factors identified for overweight or obesity in preschool–aged children under three different growth criteria.

**Interaction items**	**China criteria**			**WHO criteria**			**IOTF criteria**		
	**OR**	**95% CI**	**P**	**OR**	**95% CI**	**P**	**OR**	**95% CI**	**P**
Normal maternal BMI / Sleep duration ≥10 h	Ref.			Ref.			Ref.		
Normal maternal BMI / Sleep duration <10 h	1.19	1.01 to 1.41	0.041	1.18	0.96 to 1.45	0.116	1.17	0.95 to 1.43	0.134
Low maternal BMI / Sleep duration ≥10 h	0.70	0.53 to 0.92	0.010	0.66	0.45 to 0.96	0.028	0.76	0.54 to 1.06	0.111
Low maternal BMI / Sleep duration <10 h	1.22	0.84 to 1.78	0.288	1.08	0.68 to 1.73	0.744	1.20	0.77 to 1.88	0.419
High maternal BMI / Sleep duration ≥10 h	1.41	1.20 to 1.67	<0.001	1.48	1.20 to 1.83	<0.001	1.45	1.19 to 1.76	<0.001
High maternal BMI / Sleep duration <10 h	1.79	1.39 to 2.32	<0.001	1.85	1.38 to 2.49	<0.001	2.18	1.64 to 2.88	<0.001
Normal maternal BMI / Breastfeeding ≥10 m	Ref.			Ref.			Ref.		
Normal maternal BMI / Breastfeeding <10 m	1.18	1.01 to 1.38	0.033	1.17	0.96 to 1.42	0.117	1.31	1.09 to 1.57	0.004
Low maternal BMI / Breastfeeding ≥10 m	0.69	0.52 to 0.93	0.014	0.75	0.51 to 1.10	0.139	0.82	0.57 to 1.17	0.276
Low maternal BMI / Breastfeeding <10 m	1.06	0.77 to 1.46	0.706	0.86	0.56 to 1.32	0.482	1.06	0.71 to 1.56	0.792
High maternal BMI / Breastfeeding ≥10 m	1.52	1.28 to 1.81	<0.001	1.52	1.22 to 1.88	<0.001	1.62	1.32 to 1.99	<0.001
High maternal BMI / Breastfeeding <10 m	1.57	1.26 to 1.96	<0.001	1.78	1.37 to 2.31	<0.001	1.89	1.48 to 2.43	<0.001
Normal maternal pre–pregnancy BMI / Sleep duration ≥10 h	Ref.			Ref.			Ref.		
Normal maternal pre–pregnancy BMI / Sleep duration <10 h	1.24	1.05 to 1.46	0.010	1.19	0.97 to 1.45	0.097	1.19	0.98 to 1.45	0.082
Low maternal pre–pregnancy BMI / Sleep duration ≥10 h	0.70	0.57 to 0.85	0.001	0.66	0.50 to 0.87	0.003	0.67	0.52 to 0.87	0.002
Low maternal pre–pregnancy BMI / Sleep duration <10 h	1.28	0.95 to 1.72	0.101	1.32	0.94 to 1.87	0.113	1.37	0.98 to 1.92	0.066
High maternal pre–pregnancy BMI / Sleep duration ≥10 h	1.57	1.30 to 1.90	<0.001	1.57	1.24 to 1.99	<0.001	1.42	1.14 to 1.77	0.002
High maternal pre–pregnancy BMI / Sleep duration <10 h	1.40	1.00 to 1.95	0.050	1.39	0.94 to 2.06	0.099	1.58	1.09 to 2.28	0.015
Normal maternal pre–pregnancy BMI / Breastfeeding ≥10 m	Ref.			Ref.			Ref.		
Normal maternal pre–pregnancy BMI / Breastfeeding <10 m	1.19	1.03 to 1.39	0.021	1.19	0.99 to 1.44	0.063	1.29	1.08 to 1.54	0.005
Low maternal pre–pregnancy BMI / Breastfeeding ≥10 m	0.74	0.60 to 0.91	0.005	0.75	0.57 to 1.00	0.047	0.72	0.55 to 0.94	0.017
Low maternal pre–pregnancy BMI / Breastfeeding <10 m	0.97	0.75 to 1.26	0.828	0.97	0.70 to 1.34	0.835	1.09	0.81 to 1.49	0.547
High maternal pre–pregnancy BMI / Breastfeeding ≥10 m	1.58	1.29 to 1.93	<0.001	1.60	1.25 to 2.06	<0.001	1.52	1.21 to 1.93	<0.001
High maternal pre–pregnancy BMI / Breastfeeding <10 m	1.50	1.15 to 1.95	0.003	1.56	1.13 to 2.14	0.007	1.55	1.15 to 2.10	0.004
Sleep duration ≥10 h / Breastfeeding ≥10 m	Ref.			Ref.			Ref.		
Sleep duration <10 h / Breastfeeding ≥10 m	1.14	0.95 to 1.35	0.155	1.16	0.94 to 1.44	0.168	1.17	0.95 to 1.45	0.131
Sleep duration ≥10 h / Breastfeeding <10 m	1.06	0.91 to 1.23	0.434	1.10	0.91 to 1.33	0.319	1.17	0.98 to 1.40	0.080
Sleep duration <10 h / Breastfeeding <10 m	1.51	1.23 to 1.86	<0.001	1.41	1.11 to 1.81	0.006	1.63	1.29 to 2.06	<0.001

Abbreviations: OR, odds ratio; 95% CI, 95% confidence interval; BMI, body mass index; Ref., reference; WHO, World Health Organization; IOTF, International Obesity Task Force. P values were calculated after adjusting for age of children, sex, region, birthweight, delivery mode, maternal age, paternal age, gestational diabetes mellitus and gestational hypertension.

Low (versus normal) maternal BMI was associated with a significantly reduced risk of childhood overweight or obesity under the China criteria (OR, 95% CI, P: 0.70, 0.53 to 0.92, 0.010), whereas in the presence of short (versus long) sleep duration or short (versus long) breastfeeding this risk was weakened or reversed, albeit without statistical significance. The scenario was the same for low maternal pre–pregnancy BMI. High (versus normal) maternal BMI was significantly associated with the risk of childhood overweight or obesity under three different growth criteria, especially in the presence of short (versus long) sleep duration or short (versus long) breastfeeding. By contrast, although high (versus normal) maternal pre–pregnancy BMI was associated with a significantly increased risk of childhood overweight or obesity, the magnitude of risk was relatively weak in the presence of short (versus long) sleep duration or short (versus long) breastfeeding under three different growth criteria.

As for the interaction between sleep duration and breastfeeding duration, relative to the coexistence of long sleep duration and long breastfeeding, preschool–aged children with both short sleep duration and short breastfeeding were about 1.5 times more likely to become overweight or obese (OR, 95% CI, P: 1.51, 1.23 to 1.86, <0.001 under China criteria, 1.41, 1.11 to 1.81, 0.006 under WHO criteria, and 1.63, 1.29 to 2.06, <0.001 under IOTF criteria), but no significance was noticed for unilateral existence of short sleep duration or short breastfeeding, indicating a synergistic interaction between sleep duration and breastfeeding duration in predicting childhood overweight or obesity.

### Risk prediction nomogram model

**Figure 2:**
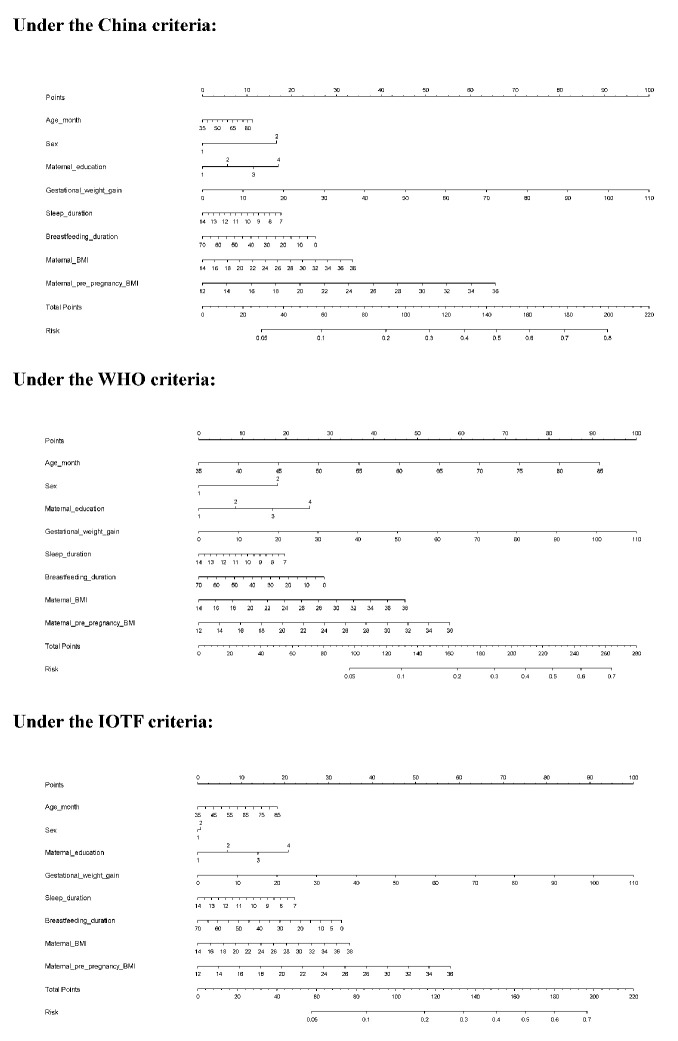
**The risk prediction nomogram models under three different growth criteria.** Abbreviations: WHO, World Health Organization; IOTF, International Obesity Task Force

To enhance practical application, a risk prediction nomogram model was created for overweight or obesity in preschool–aged children on the basis of the four significant factors identified and some conventional risk factors under three different growth criteria (Figure 2). The predictive accuracy was good, as reflected by both C–indexes which were over 80% under three different growth criteria (all P <0.001) and calibration curves (Supplementary Figure 1).

Taking the risk prediction nomogram model under the China criteria as an example: assuming a boy (17 points) aged 60 months (5 points), with maternal education of high school (12 points), GWG of 30 kg (28 points), sleeping duration of 11 hours (7.5 points), breastfeeding duration of 5 months (23 points), maternal BMI of 30 kg/m^2^ (22.5 points), and maternal pre–pregnancy BMI of 28 kg/m^2^ (44 points), the probability of childhood overweight and obesity was estimated to be 64%.

## DISCUSSION

In this large–scale cross–sectional study, we aimed to identify potential risk factors for overweight and obesity among 7222 preschool–aged children. The key finding of this study is that four significant risk factors - high maternal BMI, high maternal pre–pregnancy BMI, short breastfeeding duration, and short sleep duration, were found to be independently and consistently associated with the risk of childhood overweight or obesity under one national growth criterion from China and two widely–used international growth criteria (WHO and IOTF). Importantly, our interaction analyses revealed that the four risk factors might act in an additive manner, especially for the interaction between maternal obesity, sleep duration, and breastfeeding duration. To our knowledge, this is the first study that has interrogated the possibly interactive risk profiling of childhood overweight or obesity.

As childhood obesity is of epidemic proportions globally and impacts on the future risk of many common diseases, a great deal of attention has been paid to prevent the development of obesity, especially among preschool–aged children [17, 18]. The causes for childhood obesity have been exhaustively investigated, yet the results are not often reproducible. For example, some studies have showed that exclusive breastfeeding was a significant protective factor against overweight and obesity in children [19, 20], whereas others failed to support this claim [21–24]. Several possible reasons could account for these inconsistent findings. First, childhood obesity is a multifactorial chronic disease [25]. Besides related parental factors (such as smoking and family income), eating behaviors and dietary intake habits in infancy and childhood, as well as inherited factors, are likewise no less important [26, 27]. As the majority of epidemiological evidence currently is from cross–sectional studies, longitudinal cohorts with a comprehensive coverage of potential risk factors are encouraged. Second, residual confounding due to incomplete adjustment of measured or unmeasured covariates might exist [28, 29]. To partly address this issue, we undertook a hierarchical degree of adjustment for possible covariates, and identified four promising factors that were independently and consistently associated with the risk of childhood overweight or obesity under three different growth criteria, in line with the results of some previous studies [28–38]. Nonetheless, it seems unlikely that our findings might be interpreted by confounding. Third, it is commonly recognized that the effect of any risk factor may be small when assessed individually, and it may be more pronounced in the presence of other risk factors. Thus far, most association studies focused on risk factors individually, while disregarding other factors with small effects and overlooking possible factor–to–factor interactions. Extending the results of previous studies, we further explored the interactions between the four significant factors identified, and observed a synergistic interaction between maternal obesity, sleep duration, and breastfeeding duration in predicting the risk of overweight or obesity among preschool–aged children.

The findings of this study underscore the importance of maternal impact on offspring obesity. In fact, mothers usually act as the primary caregivers for their children and affect their behaviors and attitudes towards future life [21]. There is evidence that lower maternal education level and lower socio–economic level may lead to a worse understanding of the unhealthy weight status of children and the awareness of risk linked to obesity [30]. Maternal pre–pregnancy obesity, maternal pre–pregnancy smoking, and GWG may affect the fetus in the intrauterine environment with the ‘fuel–mediated teratogenesis’ and ‘developmental origin of health and disease’ hypotheses [39–41]. Some studies also demonstrated that relative to paternal obesity, the impact of maternal obesity on offspring obesity was remarkably strong [42–44], consistent with the findings of this present study. Besides maternal BMI, we also confirmed the significant contribution of maternal pre–pregnancy BMI to the risk of childhood overweight or obesity.

Moreover, children’s lifestyle habits also play a contributory role in the development of overweight or obesity [45]. As reported by Beebe and colleagues [45], sleep restriction in adolescents may cause increased consumption of foods with a high glycemic index, particularly desserts and sweets. Likewise, our findings indicated that per hour increase in sleep duration was significantly associated with a 5–6% decreased risk of childhood overweight or obesity, in agreement with the results of some studies [35, 46, 47]. The relation between sleep duration and childhood obesity is biologically plausible. On one hand, sleep deprivation was reported to be associated with various hormonal responses including low leptin and high endocannabinoids [48–50], and these responses may cause appetite dysregulation and further affect hunger and satiety. On the other hand, short sleep duration can activate inflammatory pathways, which can regulate the expression of genes involved in oxidative stress and metabolism [51], and further increase food and total energy intake without compensation by changes in resting metabolic rate or physical activity [52]. As an extension of previous studies, we, in this study, noticed that short sleep duration, in the presence of short breastfeeding were significantly associated with a 41–63% increased risk of childhood overweight or obesity, and this association was reinforced in the presence of high maternal BMI or high maternal pre–pregnancy BMI, indicating the joint contribution of maternal factors and offspring habits to the risk of overweight or obesity in children.

Finally, to enhance the practical application of our findings, we created a risk prediction nomogram model for childhood overweight or obesity based on statistically significant and conventional attributes under each growth criterion, and importantly this model has a decent prediction accuracy. Nonetheless, in view of multiple tests and wide confidence intervals for some comparisons, we agree that our findings should be regarded preliminary and validation in other independent groups is critical.

Despite the clear strengths of this study, including a relatively large, population–based survey on preschool–aged children, adoption of three different growth criteria, implementation of graded multivariable adjustment, and interaction explorations, several possible limitations merit special consideration. First, this study is cross–sectional in nature, precluding further comments on the cause–effect relationship. Second, only dietary–related information on the weekly intake frequencies of high-calorie foods and dining types was surveyed from study children, and it is of added interest to incorporate more dietary data such as dietary phytochemical index (DPI) [53]. Third, the data on breastfeeding and sleep duration were self–reported by parents or guardians, and hence a recall bias cannot be ruled out. Fourth, all study children are of Chinese descent and from Beijing, and thus external replication of our findings is needed.

Taken together, via an analysis of survey data from 7222 preschool–aged children and their parents or guardians, we have identified four risk factors that were independently and consistently associated with the significant risk of childhood overweight or obesity under three different growth criteria. Importantly, the four risk factors might act in an additive manner in predisposition to childhood overweight or obesity. For practical reasons, we hope the present study will not remain just another endpoint of research instead of a beginning to establish background data to further explore potential risk factors, both individually and interactively, of childhood overweight or obesity, and the possible molecular mechanisms.

## MATERIALS AND METHODS

### Study design and ethical aspects

This is a cross–sectional survey done in Beijing from September to November 2019. The conduct of this survey was reviewed and approved by the Ethics Committee of China–Japan Friendship Hospital, and was in compliance with the principles of the Declaration of Helsinki. Parents or guardians of all involved children read and signed informed consent form prior to participation.

### Study subjects

All study subjects were preschool–aged children who attended junior to senior kindergarten classes at the time this survey was conducted. Utilizing a stratified cluster random sampling strategy, out of 16 districts in Beijing, 4 districts, including 2 urban and 2 suburban ones, were selected, and within each district, 5 public or private kindergartens were selected. Self–designed questionnaires were sent to the parents or guardians of 7524 children in total, and 99.3% of them (n=7469) returned the questionnaires within the stipulated time. Children in unhealthy conditions including chronic kidney disease, hypothyroidism, congenital heart disease, or chronic respiratory diseases were excluded from the present analysis. Completed questionnaires were carefully reviewed by trained staff, and 7222 of them were deemed eligible for inclusion.

### Data collection and quality control

Questionnaires were designed to collect data from both children and their parents on the possible risk profiling of childhood obesity. From children, the data extracted included sex, date of birth, birth weight, delivery mode, breastfeeding duration, region, as well as the weekly intake frequencies of sugared beverages, sweet foods, dining out, takeout eating, cooking at home, and night meals, time of adding complementary foods, eating speed, and daily sleep duration. Body weight (to the nearest 0.1 kg) and height (to the nearest 0.1 cm) of children were measured by trained health physicians. From their parents, self–reported data on age, sex, weight, height, maternal pre–pregnancy weight, gestational weight gain, education, family income, cigarette smoking, gestational diabetes mellitus, gestational hypertension, and medical history before and during pregnancy were recorded.

Kindergarten teachers were responsible for sending the self–designed questionnaires to the parents or guardians of all children, and completed questionnaires were collected online. Data were exported from electronic questionnaires to a Microsoft Office Excel^TM^ spreadsheet, and were cross checked by trained staff. In case of missing or uncertain records, kindergarten teachers contacted parents or guardians for clarity.

### Overweight and obesity definition

In this study, one national growth criterion and two widely–used international growth criteria were employed to define childhood overweight and obesity, including the China criteria (2009), the World Health Origination (WHO) criteria (2006), and the International Obesity Task Force (IOTF) criteria (2000).

Under the China criteria [54], overweight and obesity in preschool–aged children are based on the age– and sex–specific measures, as is the case with the IOTF criteria [55].

Under the WHO criteria, overweight and obesity are defined based on body mass index (BMI) z–scores at a cutoff of 5 years old. BMI is calculated as weight divided by height squared (kg/m^2^). In detail, before 5 years old, overweight and obesity are separately defined as a BMI z–score >2 standard deviation (SD) and a BMI z–score >3 SD [56]. After 5 years old, overweight and obesity are separately defined as a BMI z–score >1 SD and a BMI z–score >2 SD [57].

### Definitions of the other characteristics

Dietary–related information from study children included the weekly intake frequencies of sugared beverages, sweet foods, dining out, takeout eating, cooking at home, and night meals, as well as eating speed. Sugared beverages included drinks with high amounts of added sugar (e.g. Coca–Cola, carbonated drinks, milk tea, and black tea). Sweet foods covered foods containing high sugar (e.g. cakes, sugars, desserts, and chocolates). Dining out referred to eating in restaurants or snack bars. Takeout eating meant eating at home the foods cooked by restaurants or snack bars. Night meals were the same as the extra meals in a day, defined as eating some foods within 2 hours before bedtime. Weekly intake frequency was graded as every day, often (3–5 times), occasional (1–2 times) or none or once in a while. Eating speed was calculated as the average time of breakfast, lunch, and supper.

For children, sleep duration was calculated as the sum of both sleep time on work days × 5 and sleep time on weekends × 2 divided by 7. Breastfeeding duration and time of adding complementary foods were recorded in months.

Parental BMI and maternal pre–pregnancy BMI were derived from self–reported height and weight. Based on pre–pregnancy BMI, mothers were classified as underweight (<18.5 kg/m^2^), normal weight (18.5–24 kg/m^2^), and overweight/obesity (≥24 kg/m^2^) [15]. Gestational weight gain (GWG) was calculated by subtracting maternal pre–pregnancy weight from maternal weight at delivery, and it was grouped into inadequate, adequate, and excessive GWG according to the recommendations of the Institute of Medicine (2009) [58]. Specifically, adequate GWG is defined as a weight gain of 12.5–18.0 kg in underweight mothers, 11.5–16.0 kg in normal–weight mothers, 7.0–11.5 kg in overweight mothers, and 5.0–9.0 kg in obese mothers. Within–group weight gains less than the lower limits and greater than the upper limits of above ranges are defined as inadequate GWG and excessive GWG, respectively.

Education level was categorized as graduate degree or above, college (or equivalent) degree, high school (or equivalent) degree, or middle school degree or below. Family income level (RMB per year) was categorized as ≥500,000, 200,000–500,000, 100,000–200,000 or <100,000. Gestational diabetes mellitus and gestational hypertension, diagnosed by doctors from second–class or above hospitals, were recorded. Maternal pregnancy smoking was defined as smoking and non–smoking. Maternal pre–pregnancy smoking and paternal smoking were grouped into never smoking and ever (current or former) smoking. Delivery mode included natural birth and caesarean section.

### Statistical analysis

All study children were grouped into the non–overweight group and the overweight/obesity group according to three different growth criteria for childhood overweight and obesity definition. The distributions of continuous variables were assessed for normality by use of the skewness and kurtosis test. Skewed continuous variables are expressed as median (interquartile range), and normally-distributed variables as mean (SD). Categorical variables are expressed as count (percentage). Between-group comparisons of baseline characteristics were performed using the χ^2^ test, t test, or rank–sum test, where appropriate. To identify statistically significant risk factors for childhood overweight or obesity, Logistic regression analyses were first done without considering any confounders, and then adjusting for age of children, sex, and region, and additionally for birthweight, delivery mode, maternal age, paternal age, gestational diabetes mellitus, and gestational hypertension in multivariable adjustment models under three different growth criteria. Effect–size estimates are expressed as odds ratio (OR) and 95% confidence interval (95% CI). Finally, four risk factors, including high maternal BMI, high maternal pre–pregnancy BMI, short breastfeeding duration, and short sleep duration were significantly and independently associated with the risk of childhood overweight or obesity, and importantly significance persisted under three different growth criteria.

Predictive accuracy gained by adding the four significant risk factors to the basic model (including age of children, sex, region, birthweight, time of adding complementary foods, and the weekly intake frequencies of sugared beverages, sweet foods, dining out, takeout eating, cooking at home and night meals, as well as eating speed, maternal age, paternal age, paternal BMI, gestational weight gain, paternal education, maternal education, family income, gestational diabetes mellitus, gestational hypertension, maternal pre–pregnancy smoking, maternal pregnancy smoking, paternal smoking and delivery mode) was appraised from both calibration and discrimination aspects. From the calibration aspect, Akaike information criterion (AIC) and Bayesian information criterion (BIC), as well as the −2 log likelihood ratio test were used to appraise how closely the prediction probability by adding the four significant risk factors reflected the actual observed risk and global fit of modified risk model. From the discrimination aspect, net reclassification improvement (NRI) and integrated discrimination improvement (IDI) [59, 60] were used to see whether the addition of the four significant risk factors can differentiate preschool–aged children in the non–overweight group or the overweight/obesity group. Moreover, the net benefits for the addition of the four significant risk factors were also inspected by decision curve analysis [61].

Finally, a risk prediction nomogram model for childhood overweight or obesity was created under three different growth criteria, and predictive accuracy was reflected by concordance index (C–index), defined as the area under the receiver operating characteristics curve. The nomogram and accuracy assessment were implemented by the R programming environment (version 3.5.2) “rms” package.

Statistical analyses were completed using the STATA software (version 14.0, Stata Corp, TX) unless otherwise indicated. Two–sided P value less than 0.05 was considered statistically significant.

## Supplementary Material

Supplementary Figure 1

Supplementary Table 1

## References

[1] Lobstein T, Jackson-Leach R, Moodie ML, Hall KD, Gortmaker SL, Swinburn BA, James WP, Wang Y, McPherson K. Child and adolescent obesity: part of a bigger picture. Lancet. 2015; 385:2510–20. 10.1016/S0140-6736(14)61746-3 25703114 PMC4594797

[2] Lobstein T, Baur L, Uauy R, and IASO International Obesity TaskForce. Obesity in children and young people: a crisis in public health. Obes Rev. 2004 (Suppl 1); 5:4–104. 10.1111/j.1467-789X.2004.00133.x 15096099

[3] Ng M, Fleming T, Robinson M, Thomson B, Graetz N, Margono C, Mullany EC, Biryukov S, Abbafati C, Abera SF, Abraham JP, Abu-Rmeileh NM, Achoki T, et al. Global, regional, and national prevalence of overweight and obesity in children and adults during 1980-2013: a systematic analysis for the global burden of disease study 2013. Lancet. 2014; 384:766–81. 10.1016/S0140-6736(14)60460-8 24880830 PMC4624264

[4] Jia P, Xue H, Zhang J, Wang Y. Time trend and demographic and geographic disparities in childhood obesity prevalence in China-evidence from twenty years of longitudinal data. Int J Environ Res Public Health. 2017; 14:369. 10.3390/ijerph14040369 28362361 PMC5409570

[5] Daniels SR. The consequences of childhood overweight and obesity. Future Child. 2006; 16:47–67. 10.1353/foc.2006.0004 16532658

[6] Jia P, Li M, Xue H, Lu L, Xu F, Wang Y. School environment and policies, child eating behavior and overweight/obesity in urban China: the childhood obesity study in China megacities. Int J Obes (Lond). 2017; 41:813–19. 10.1038/ijo.2017.2 28074059

[7] Krul M, van der Wouden JC, Schellevis FG, van Suijlekom-Smit LW, Koes BW. Musculoskeletal problems in overweight and obese children. Ann Fam Med. 2009; 7:352–56. 10.1370/afm.1005 19597173 PMC2713163

[8] Must A, Jacques PF, Dallal GE, Bajema CJ, Dietz WH. Long-term morbidity and mortality of overweight adolescents. A follow-up of the harvard growth study of 1922 to 1935. N Engl J Med. 1992; 327:1350–55. 10.1056/NEJM199211053271904 1406836

[9] Abdullah A, Wolfe R, Stoelwinder JU, de Courten M, Stevenson C, Walls HL, Peeters A. The number of years lived with obesity and the risk of all-cause and cause-specific mortality. Int J Epidemiol. 2011; 40:985–96. 10.1093/ije/dyr018 21357186

[10] Park MH, Falconer C, Viner RM, Kinra S. The impact of childhood obesity on morbidity and mortality in adulthood: a systematic review. Obes Rev. 2012; 13:985–1000. 10.1111/j.1467-789X.2012.01015.x 22731928

[11] Oken E. Maternal and child obesity: the causal link. Obstet Gynecol Clin North Am. 2009; 36:361–77. 10.1016/j.ogc.2009.03.007 19501319

[12] Mirza N, Phan TL, Tester J, Fals A, Fernandez C, Datto G, Estrada E, Eneli I. A narrative review of medical and genetic risk factors among children age 5 and younger with severe obesity. Child Obes. 2018; 14:443–52. 10.1089/chi.2017.0350 29791184 PMC6157342

[13] Hills AP, Andersen LB, Byrne NM. Physical activity and obesity in children. Br J Sports Med. 2011; 45:866–70. 10.1136/bjsports-2011-090199 21836171

[14] Liu S, Zhang J, Ma J, Shang Y, Ma Y, Zhang X, Wang S, Yuan Y, Deng X, Niu W, Zhang Z. Synergistic interaction between bedtime and eating speed in predicting overweight and obesity in Chinese preschool-aged children. Aging (Albany NY). 2019; 11:2127–37. 10.18632/aging.101906 30978174 PMC6503874

[15] Zhou BF, and Cooperative Meta-Analysis Group of the Working Group on Obesity in China. Predictive values of body mass index and waist circumference for risk factors of certain related diseases in Chinese adults—study on optimal cut-off points of body mass index and waist circumference in Chinese adults. Biomed Environ Sci. 2002; 15:83–96. 12046553

[16] Paruthi S, Brooks LJ, D’Ambrosio C, Hall WA, Kotagal S, Lloyd RM, Malow BA, Maski K, Nichols C, Quan SF, Rosen CL, Troester MM, Wise MS. Recommended amount of sleep for pediatric populations: a consensus statement of the American academy of sleep medicine. J Clin Sleep Med. 2016; 12:785–86. 10.5664/jcsm.5866 27250809 PMC4877308

[17] Gehanno JF, Gehanno B, Schuers M, Grosjean J, Rollin L. Analysis of publication trends in childhood obesity research in PubMed since 1945. Child Obes. 10.1089/chi.2018.0276 30855177

[18] Rudolf MC, Levine R, Feltbower R, Connor A, Robinson M. The TRENDS project: development of a methodology to reliably monitor the obesity epidemic in childhood. Arch Dis Child. 2006; 91:309–911. 10.1136/adc.2005.078915 16354712 PMC2065996

[19] Lee JW, Lee M, Lee J, Kim YJ, Ha E, Kim HS. The protective effect of exclusive breastfeeding on overweight/obesity in children with high birth weight. J Korean Med Sci. 2019; 34:e85. 10.3346/jkms.2019.34.e85 30886551 PMC6417996

[20] Wang L, Collins C, Ratliff M, Xie B, Wang Y. Breastfeeding reduces childhood obesity risks. Child Obes. 2017; 13:197–204. 10.1089/chi.2016.0210 28398851

[21] Reilly JJ, Armstrong J, Dorosty AR, Emmett PM, Ness A, Rogers I, Steer C, Sherriff A, and Avon Longitudinal Study of Parents and Children Study Team. Early life risk factors for obesity in childhood: cohort study. BMJ. 2005; 330:1357. 10.1136/bmj.38470.670903.E0 15908441 PMC558282

[22] Casazza K, Fontaine KR, Astrup A, Birch LL, Brown AW, Bohan Brown MM, Durant N, Dutton G, Foster EM, Heymsfield SB, McIver K, Mehta T, Menachemi N, et al. Myths, presumptions, and facts about obesity. N Engl J Med. 2013; 368:446–54. 10.1056/NEJMsa1208051 23363498 PMC3606061

[23] Kramer MS, Matush L, Vanilovich I, Platt RW, Bogdanovich N, Sevkovskaya Z, Dzikovich I, Shishko G, Collet JP, Martin RM, Davey Smith G, Gillman MW, Chalmers B, et al, and PROBIT Study Group. Effects of prolonged and exclusive breastfeeding on child height, weight, adiposity, and blood pressure at age 6.5 Y: evidence from a large randomized trial. Am J Clin Nutr. 2007; 86:1717–21. 10.1093/ajcn/86.5.1717 18065591

[24] Burdette HL, Whitaker RC, Hall WC, Daniels SR. Breastfeeding, introduction of complementary foods, and adiposity at 5 Y of age. Am J Clin Nutr. 2006; 83:550–58. 10.1093/ajcn.83.3.550 16522900

[25] Di Cesare M, Sorić M, Bovet P, Miranda JJ, Bhutta Z, Stevens GA, Laxmaiah A, Kengne AP, Bentham J. The epidemiological burden of obesity in childhood: a worldwide epidemic requiring urgent action. BMC Med. 2019; 17:212. 10.1186/s12916-019-1449-8 31760948 PMC6876113

[26] Parsons TJ, Power C, Logan S, Summerbell CD. Childhood predictors of adult obesity: a systematic review. Int J Obes Relat Metab Disord. 1999 (Suppl 8); 23:S1–107. 10641588

[27] Albataineh SR, Badran EF, Tayyem RF. Dietary factors and their association with childhood obesity in the middle east: a systematic review. Nutr Health. 2019; 25:53–60. 10.1177/0260106018803243 30282516

[28] Bider-Canfield Z, Martinez MP, Wang X, Yu W, Bautista MP, Brookey J, Page KA, Buchanan TA, Xiang AH. Maternal obesity, gestational diabetes, breastfeeding and childhood overweight at age 2 years. Pediatr Obes. 2017; 12:171–78. 10.1111/ijpo.12125 26956226

[29] Robinson SM, Crozier SR, Harvey NC, Barton BD, Law CM, Godfrey KM, Cooper C, Inskip HM. Modifiable early-life risk factors for childhood adiposity and overweight: an analysis of their combined impact and potential for prevention. Am J Clin Nutr. 2015; 101:368–75. 10.3945/ajcn.114.094268 25646335 PMC4307207

[30] Parrino C, Vinciguerra F, La Spina N, Romeo L, Tumminia A, Baratta R, Squatrito S, Vigneri R, Frittitta L. Influence of early-life and parental factors on childhood overweight and obesity. J Endocrinol Invest. 2016; 39:1315–21. 10.1007/s40618-016-0501-1 27312861

[31] Kaul P, Bowker SL, Savu A, Yeung RO, Donovan LE, Ryan EA. Association between maternal diabetes, being large for gestational age and breast-feeding on being overweight or obese in childhood. Diabetologia. 2019; 62:249–58. 10.1007/s00125-018-4758-0 30421138

[32] Kain J, Leyton B, Baur L, Lira M, Corvalán C. Demographic, social and health-related variables that predict normal-weight preschool children having overweight or obesity when entering primary education in Chile. Nutrients. 2019; 11:1277. 10.3390/nu11061277 31195698 PMC6627860

[33] Hu Z, Tylavsky FA, Han JC, Kocak M, Fowke JH, Davis RL, Lewinn K, Bush NR, Zhao Q. Maternal metabolic factors during pregnancy predict early childhood growth trajectories and obesity risk: the CANDLE Study. Int J Obes (Lond). 2019; 43:1914–1922. 10.1038/s41366-019-0326-z 30705389 PMC6669102

[34] Felső R, Lohner S, Hollódy K, Erhardt É, Molnár D. Relationship between sleep duration and childhood obesity: systematic review including the potential underlying mechanisms. Nutr Metab Cardiovasc Dis. 2017; 27:751–61. 10.1016/j.numecd.2017.07.008 28818457

[35] Wang F, Liu H, Wan Y, Li J, Chen Y, Zheng J, Huang T, Li D. Sleep duration and overweight/obesity in preschool-aged children: a prospective study of up to 48,922 children of the jiaxing birth cohort. Sleep. 2016; 39:2013–19. 10.5665/sleep.6234 27568808 PMC5070755

[36] McDonald L, Wardle J, Llewellyn CH, van Jaarsveld CH, Fisher A. Predictors of shorter sleep in early childhood. Sleep Med. 2014; 15:536–40. 10.1016/j.sleep.2014.01.005 24726571 PMC4038745

[37] Weng SF, Redsell SA, Swift JA, Yang M, Glazebrook CP. Systematic review and meta-analyses of risk factors for childhood overweight identifiable during infancy. Arch Dis Child. 2012; 97:1019–26. 10.1136/archdischild-2012-302263 23109090 PMC3512440

[38] Woo Baidal JA, Locks LM, Cheng ER, Blake-Lamb TL, Perkins ME, Taveras EM. Risk factors for childhood obesity in the first 1,000 days: a systematic review. Am J Prev Med. 2016; 50:761–79. 10.1016/j.amepre.2015.11.012 26916261

[39] Freinkel N. Banting lecture 1980. Of pregnancy and progeny. Diabetes. 1980; 29:1023–35. 10.2337/diab.29.12.1023 7002669

[40] Barker DJ. The origins of the developmental origins theory. J Intern Med. 2007; 261:412–17. 10.1111/j.1365-2796.2007.01809.x 17444880

[41] Alfaradhi MZ, Ozanne SE. Developmental programming in response to maternal overnutrition. Front Genet. 2011; 2:27. 10.3389/fgene.2011.00027 22303323 PMC3268582

[42] McLoone P, Morrison DS. Risk of child obesity from parental obesity: analysis of repeat national cross-sectional surveys. Eur J Public Health. 2014; 24:186–90. 10.1093/eurpub/cks175 23254271

[43] Lazzeri G, Pammolli A, Pilato V, Giacchi MV. Relationship between 8/9-yr-old school children BMI, parents’ BMI and educational level: a cross sectional survey. Nutr J. 2011; 10:76. 10.1186/1475-2891-10-76 21771312 PMC3160354

[44] Linabery AM, Nahhas RW, Johnson W, Choh AC, Towne B, Odegaard AO, Czerwinski SA, Demerath EW. Stronger influence of maternal than paternal obesity on infant and early childhood body mass index: the fels longitudinal study. Pediatr Obes. 2013; 8:159–69. 10.1111/j.2047-6310.2012.00100.x 23042783 PMC3765070

[45] Beebe DW, Simon S, Summer S, Hemmer S, Strotman D, Dolan LM. Dietary intake following experimentally restricted sleep in adolescents. Sleep. 2013; 36:827–34. 10.5665/sleep.2704 23729925 PMC3649825

[46] Watanabe E, Lee JS, Mori K, Kawakubo K. Clustering patterns of obesity-related multiple lifestyle behaviours and their associations with overweight and family environments: a cross-sectional study in Japanese preschool children. BMJ Open. 2016; 6:e012773. 10.1136/bmjopen-2016-012773 27815299 PMC5128936

[47] Miller MA, Kruisbrink M, Wallace J, Ji C, Cappuccio FP. Sleep duration and incidence of obesity in infants, children, and adolescents: a systematic review and meta-analysis of prospective studies. Sleep. 2018; 41:10.1093/sleep/zsy018. 10.1093/sleep/zsy018 29401314

[48] Spiegel K, Tasali E, Penev P, Van Cauter E. Brief communication: sleep curtailment in healthy young men is associated with decreased leptin levels, elevated ghrelin levels, and increased hunger and appetite. Ann Intern Med. 2004; 141:846–50. 10.7326/0003-4819-141-11-200412070-00008 15583226

[49] Taheri S, Lin L, Austin D, Young T, Mignot E. Short sleep duration is associated with reduced leptin, elevated ghrelin, and increased body mass index. PLoS Med. 2004; 1:e62. 10.1371/journal.pmed.0010062 15602591 PMC535701

[50] Cedernaes J, Fanelli F, Fazzini A, Pagotto U, Broman JE, Vogel H, Dickson SL, Schiöth HB, Benedict C. Sleep restriction alters plasma endocannabinoids concentrations before but not after exercise in humans. Psychoneuroendocrinology. 2016; 74:258–68. 10.1016/j.psyneuen.2016.09.014 27689899

[51] Möller-Levet CS, Archer SN, Bucca G, Laing EE, Slak A, Kabiljo R, Lo JC, Santhi N, von Schantz M, Smith CP, Dijk DJ. Effects of insufficient sleep on circadian rhythmicity and expression amplitude of the human blood transcriptome. Proc Natl Acad Sci USA. 2013; 110:E1132–41. 10.1073/pnas.1217154110 23440187 PMC3607048

[52] Capers PL, Fobian AD, Kaiser KA, Borah R, Allison DB. A systematic review and meta-analysis of randomized controlled trials of the impact of sleep duration on adiposity and components of energy balance. Obes Rev. 2015; 16:771–82. 10.1111/obr.12296 26098388 PMC4532553

[53] Eslami O, Khoshgoo M, Shidfar F. Dietary phytochemical index and overweight/obesity in children: a cross-sectional study. BMC Res Notes. 2020; 13:132. 10.1186/s13104-020-04979-6 32138761 PMC7059655

[54] Li H, Ji CY, Zong XN, Zhang YQ. [Body mass index growth curves for Chinese children and adolescents aged 0 to 18 years]. Zhonghua Er Ke Za Zhi. 2009; 47:493–98. 19951508

[55] Cole TJ, Bellizzi MC, Flegal KM, Dietz WH. Establishing a standard definition for child overweight and obesity worldwide: international survey. BMJ. 2000; 320:1240–43. 10.1136/bmj.320.7244.1240 10797032 PMC27365

[56] The Coordinating Team in the Department of Nutrition for Health and Development of the World Health Organization. WHO Child Growth Standards. World Health Organization (ISBN 92 4 154693 X). 2006:260–295.

[57] Obesity and Overweight. https://www.who.int/newsroom/fact-sheets/detail/obesity-and-overweight.

[58] (2009). In: Rasmussen KM, Yaktine AL, eds. Weight Gain During Pregnancy: Reexamining the Guidelines. (Washington (DC). 20669500

[59] Pencina MJ, D’Agostino RB Sr, D’Agostino RB Jr, Vasan RS. Evaluating the added predictive ability of a new marker: from area under the ROC curve to reclassification and beyond. Stat Med. 2008; 27:157–72. 10.1002/sim.2929 17569110

[60] Pencina MJ, D’Agostino RB, Vasan RS. Statistical methods for assessment of added usefulness of new biomarkers. Clin Chem Lab Med. 2010; 48:1703–11. 10.1515/CCLM.2010.340 20716010 PMC3155999

[61] Vickers AJ, Elkin EB. Decision curve analysis: a novel method for evaluating prediction models. Med Decis Making. 2006; 26:565–74. 10.1177/0272989X06295361 17099194 PMC2577036

