# Supplementary material for: Interaction effects of significant risk factors on overweight or obesity among 7222 preschool–aged children from Beijing
Source: Aging (Albany NY). 2020 Aug 3;12(15):15462–77. doi: 10.18632/aging.103701 (PMC7467379; doi:10.18632/aging.103701)
Supplement: Supplementary Figure 1 [file aging-12-103701-s002..pdf]

SUPPLEMENTARY FIGURE

Under the China criteria:

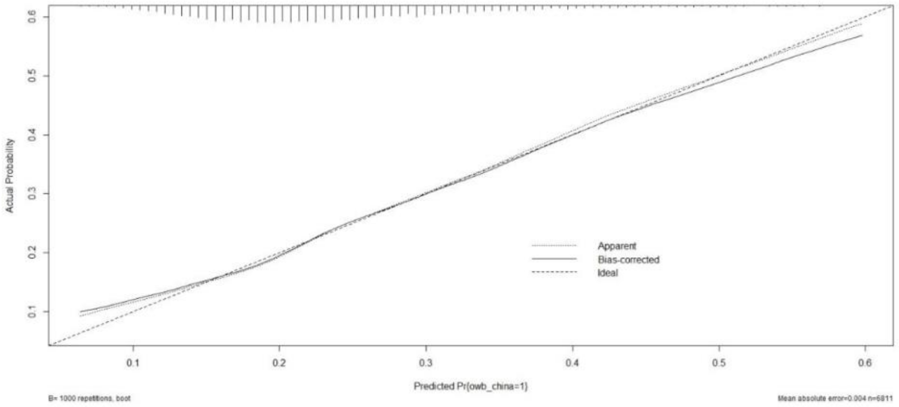

Under the WHO criteria:

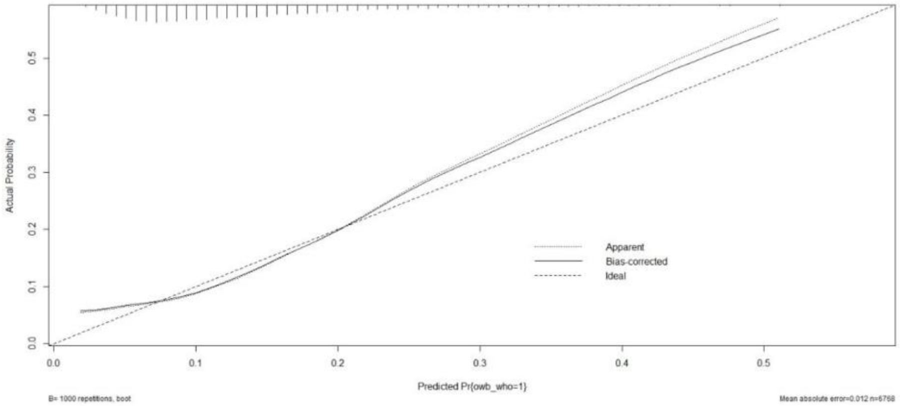

Under the IOTF criteria:

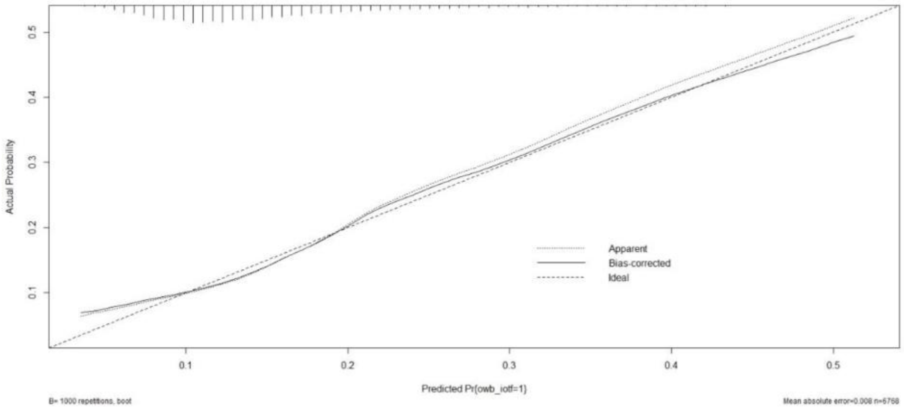

Supplementary Figure 1. The calibration curve for each nomogram model under three different growth criteria.
