# Supplementary material for: Interaction effects of significant risk factors on overweight or obesity among 7222 preschool–aged children from Beijing
Source: Aging (Albany NY). 2020 Aug 3;12(15):15462–77. doi: 10.18632/aging.103701 (PMC7467379; doi:10.18632/aging.103701)
Supplement: Supplementary Table 1 [file aging-12-103701-s001..docx]

Supplementary Table

Supplementary Table 1. The baseline characteristics of study children under three different growth criteria.

| **Characteristics** | **China criteria** | | | **WHO criteria** | | | **IOTF criteria** | | |
| --- | --- | --- | --- | --- | --- | --- | --- | --- | --- |
|  | **Non-overweight** | **Overweight/obesity** | **P** | **Non-overweight** | **Overweight/obesity** | **P** | **Non-overweight** | **Overweight/obesity** | **P** |
|  | **(n=5497)** | **(n=1725)** |  | **(n=6243)** | **(n=979)** |  | **(n=6145)** | **(n=1077)** |  |
| ***From children*** |  |  |  |  |  |  |  |  |  |
| Age |  |  |  |  |  |  |  |  |  |
| ≤5 years | 3344 (60.83%) | 998 (57.86%) | 0.028 | 3961 (63.45%) | 381 (38.92%) | <0.001 | 3745 (60.94%) | 597 (55.43%) | 0.001 |
| >5 years | 2153 (39.17%) | 727 (42.14%) |  | 2282 (36.55%) | 598 (61.08%) |  | 2400 (39.06%) | 480 (44.57%) |  |
| Age (m) | 56.91 (44.9, 63.95) | 57.86 (45.99, 64.74) | 0.004 | 56.38 (44.67, 63.29) | 62.53 (52.24, 67.86) | <0.001 | 56.91 (44.9, 63.88) | 58.68 (46.81, 65.92) | <0.001 |
| Males | 2614 (47.55%) | 1007 (58.38%) | <0.001 | 3040 (48.69%) | 581 (59.35%) | <0.001 | 3074 (50.02%) | 547 (50.79%) | 0.643 |
| Region |  |  |  |  |  |  |  |  |  |
| Urban area | 3101 (56.41%) | 842 (48.81%) | <0.001 | 3492 (55.93%) | 451 (46.07%) | <0.001 | 3443 (56.03%) | 500 (46.43%) | <0.001 |
| Suburban area | 2396 (43.59%) | 883 (51.19%) |  | 2751 (44.07%) | 528 (53.93%) |  | 2702 (43.97%) | 577 (53.57%) |  |
| BMI (kg/m^2^) | 14.9 (14.11, 15.66) | 17.94 (17.15, 19.66) | <0.001 | 15.1 (14.24, 15.99) | 19.2 (17.98, 21.7) | <0.001 | 15.08 (14.24, 15.94) | 18.92 (18.06, 21.36) | <0.001 |
| Birthweight (g) | 3331.5 (3030, 3600) | 3450 (3120, 3750) | <0.001 | 3350 (3050, 3630) | 3430 (3100, 3756) | <0.001 | 3350 (3050, 3630) | 3430 (3100, 3750) | <0.001 |
| Sleep duration | 10.57 (10, 11.29) | 10.29 (9.57, 11.29) | 1.999 | 10.57 (10, 11.29) | 10.14 (9.29, 11.29) | 1.999 | 10.57 (10, 11.29) | 10.29 (9.29, 11.29) | 1.999 |
| Breastfeeding duration (m) | 12 (7, 18) | 12 (6, 18) | 1.999 | 12 (7, 18) | 12 (6, 18) | 1.999 | 12 (7, 18) | 12 (6, 18) | 1.999 |
| Time of adding complementary food (m) | 6 (6, 6) | 6 (6, 6) | 0.008 | 6 (6, 6) | 6 (6, 7) | 0.062 | 6 (6, 6) | 6 (6, 7) | 0.142 |
| Weekly intake frequency of sugared beverages |  |  |  |  |  |  |  |  |  |
| None or once in a while | 4337 (78.9%) | 1283 (74.4%) | <0.001 | 4911 (78.7%) | 709 (72.4%) | <0.001 | 4833 (78.6%) | 787 (73.1%) | <0.001 |
| Occasional (1–2 times) | 993 (18.1%) | 371 (21.5%) |  | 1141 (18.3%) | 223 (22.8%) |  | 1123 (18.3%) | 241 (22.4%) |  |
| Often (3–5 times) | 149 (2.7%) | 58 (3.4%) |  | 167 (2.7%) | 40 (4.1%) |  | 167 (2.7%) | 40 (3.7%) |  |
| Every day | 18 (0.3%) | 13 (0.8%) |  | 24 (0.4%) | 7 (0.7%) |  | 22 (0.4%) | 9 (0.8%) |  |
| Weekly intake frequency of sweet foods |  |  |  |  |  |  |  |  |  |
| None or once in a while | 1222 (22.2%) | 408 (23.7%) | 0.105 | 1401 (22.4%) | 229 (23.4%) | 0.066 | 1381 (22.5%) | 249 (23.1%) | 0.196 |
| Occasional (1–2 times) | 2642 (48.1%) | 841 (48.8%) |  | 2991 (47.9%) | 492 (50.3%) |  | 2950 (48%) | 533 (49.5%) |  |
| Often (3–5 times) | 1253 (22.8%) | 383 (22.2%) |  | 1425 (22.8%) | 211 (21.6%) |  | 1396 (22.7%) | 240 (22.3%) |  |
| Every day | 380 (6.9%) | 93 (5.4%) |  | 426 (6.8%) | 47 (4.8%) |  | 418 (6.8%) | 55 (5.1%) |  |
| Weekly intake frequency of dining out |  |  |  |  |  |  |  |  |  |
| None or once in a while | 2020 (36.7%) | 667 (38.7%) | 0.059 | 2311 (37%) | 376 (38.4%) | 0.727 | 2281 (37.1%) | 406 (37.7%) | 0.123 |
| Occasional (1–2 times) | 3213 (58.5%) | 970 (56.2%) |  | 3624 (58%) | 559 (57.1%) |  | 3567 (58%) | 616 (57.2%) |  |
| Often (3–5 times) | 255 (4.6%) | 80 (4.6%) |  | 294 (4.7%) | 41 (4.2%) |  | 286 (4.7%) | 49 (4.5%) |  |
| Every day | 9 (0.2%) | 8 (0.5%) |  | 14 (0.2%) | 3 (0.3%) |  | 11 (0.2%) | 6 (0.6%) |  |
| Weekly intake frequency of takeout eating |  |  |  |  |  |  |  |  |  |
| None or once in a while | 4095 (74.5%) | 1277 (74%) | 0.834 | 4653 (74.5%) | 719 (73.4%) | 0.468 | 4575 (74.5%) | 797 (74%) | 0.873 |
| Occasional (1–2 times) | 1288 (23.4%) | 407 (23.6%) |  | 1451 (23.2%) | 244 (24.9%) |  | 1437 (23.4%) | 258 (24%) |  |
| Often (3–5 times) | 108 (2%) | 38 (2.2%) |  | 131 (2.1%) | 15 (1.5%) |  | 126 (2.1%) | 20 (1.9%) |  |
| Every day | 6 (0.1%) | 3 (0.2%) |  | 8 (0.1%) | 1 (0.1%) |  | 7 (0.1%) | 2 (0.2%) |  |
| Weekly intake frequency of cooking at home |  |  |  |  |  |  |  |  |  |
| None or once in a while | 97 (1.8%) | 29 (1.7%) | 0.362 | 108 (1.7%) | 18 (1.8%) | 0.270 | 106 (1.7%) | 20 (1.9%) | 0.035 |
| Occasional (1–2 times) | 432 (7.9%) | 156 (9%) |  | 495 (7.9%) | 93 (9.5%) |  | 480 (7.8%) | 108 (10%) |  |
| Often (3–5 times) | 1924 (35%) | 578 (33.5%) |  | 2182 (35%) | 320 (32.7%) |  | 2159 (35.1%) | 343 (31.8%) |  |
| Every day | 3044 (55.4%) | 962 (55.8%) |  | 3458 (55.4%) | 548 (56%) |  | 3400 (55.3%) | 606 (56.3%) |  |
| Weekly intake frequency of night meals |  |  |  |  |  |  |  |  |  |
| None or once in a while | 3323 (60.5%) | 1033 (59.9%) | 0.059 | 3759 (60.2%) | 597 (61%) | 0.096 | 3717 (60.5%) | 639 (59.3%) | 0.003 |
| Occasional (1–2 times) | 1213 (22.1%) | 420 (24.3%) |  | 1393 (22.3%) | 240 (24.5%) |  | 1350 (22%) | 283 (26.3%) |  |
| Often (3–5 times) | 636 (11.6%) | 193 (11.2%) |  | 732 (11.7%) | 97 (9.9%) |  | 720 (11.7%) | 109 (10.1%) |  |
| Every day | 325 (5.9%) | 79 (4.6%) |  | 359 (5.8%) | 45 (4.6%) |  | 358 (5.8%) | 46 (4.3%) |  |
| Breakfast time |  |  |  |  |  |  |  |  |  |
| <10 min | 962 (17.5%) | 341 (19.8%) | 0.003 | 1094 (17.5%) | 209 (21.3%) | 0.003 | 1079 (17.6%) | 224 (20.8%) | 0.054 |
| 10–20 min | 3023 (55%) | 982 (56.9%) |  | 3452 (55.3%) | 553 (56.5%) |  | 3411 (55.5%) | 594 (55.2%) |  |
| 20–30 min | 1342 (24.4%) | 369 (21.4%) |  | 1513 (24.2%) | 198 (20.2%) |  | 1475 (24%) | 236 (21.9%) |  |
| 30–40 min | 141 (2.6%) | 28 (1.6%) |  | 152 (2.4%) | 17 (1.7%) |  | 149 (2.4%) | 20 (1.9%) |  |
| 40–50 min | 29 (0.5%) | 5 (0.3%) |  | 32 (0.5%) | 2 (0.2%) |  | 31 (0.5%) | 3 (0.3%) |  |
| Lunch time |  |  |  |  |  |  |  |  |  |
| <10 min | 253 (4.6%) | 105 (6.1%) | <0.001 | 286 (4.6%) | 72 (7.4%) | <0.001 | 282 (4.6%) | 76 (7.1%) | <0.001 |
| 10–20 min | 2350 (42.8%) | 820 (47.5%) |  | 2703 (43.3%) | 467 (47.7%) |  | 2666 (43.4%) | 504 (46.8%) |  |
| 20–30 min | 2346 (42.7%) | 680 (39.4%) |  | 2654 (42.5%) | 372 (38%) |  | 2607 (42.4%) | 419 (38.9%) |  |
| 30–40 min | 461 (8.4%) | 106 (6.1%) |  | 507 (8.1%) | 60 (6.1%) |  | 501 (8.2%) | 66 (6.1%) |  |
| 40–50 min | 87 (1.6%) | 14 (0.8%) |  | 93 (1.5%) | 8 (0.8%) |  | 89 (1.4%) | 12 (1.1%) |  |
| Supper time |  |  |  |  |  |  |  |  |  |
| <10 min | 286 (5.2%) | 122 (7.1%) | <0.001 | 330 (5.3%) | 78 (8%) | <0.001 | 324 (5.3%) | 84 (7.8%) | <0.001 |
| 10–20 min | 2312 (42.1%) | 804 (46.6%) |  | 2655 (42.5%) | 461 (47.1%) |  | 2617 (42.6%) | 499 (46.3%) |  |
| 20–30 min | 2329 (42.4%) | 679 (39.4%) |  | 2636 (42.2%) | 372 (38%) |  | 2590 (42.1%) | 418 (38.8%) |  |
| 30–40 min | 474 (8.6%) | 105 (6.1%) |  | 519 (8.3%) | 60 (6.1%) |  | 514 (8.4%) | 65 (6%) |  |
| 40–50 min | 96 (1.7%) | 15 (0.9%) |  | 103 (1.6%) | 8 (0.8%) |  | 100 (1.6%) | 11 (1%) |  |
| ***From parents*** ***or guardians*** |  |  |  |  |  |  |  |  |  |
| Maternal BMI (kg/m^2^) | 21.3 (19.59, 23.44) | 22.04 (20.2, 24.46) | <0.001 | 21.45 (19.68, 23.44) | 22.08 (20.2, 24.61) | <0.001 | 21.37 (19.65, 23.44) | 22.29 (20.2, 24.61) | <0.001 |
| Paternal BMI (kg/m^2^) | 24.49 (22.72, 26.83) | 25.69 (23.55, 28.36) | <0.001 | 24.64 (22.84, 26.89) | 25.71 (23.55, 28.08) | <0.001 | 24.62 (22.84, 26.87) | 25.71 (23.51, 28.41) | <0.001 |
| Maternal pre–pregnancy BMI (kg/m^2^) | 20.43 (18.82, 22.31) | 21.26 (19.43, 23.44) | <0.001 | 20.55 (18.9, 22.49) | 21.23 (19.43, 23.44) | <0.001 | 20.5 (18.87, 22.49) | 21.26 (19.49, 23.44) | <0.001 |
| Gestational weight gain | 15 (10, 18) | 15 (11, 20) | <0.001 | 15 (10, 19) | 15 (11, 20) | <0.001 | 15 (10, 19) | 15 (11, 20) | <0.001 |
| Paternal education |  |  |  |  |  |  |  |  |  |
| Graduate degree or above | 1398 (25.43%) | 359 (20.81%) | <0.001 | 1579 (25.29%) | 178 (18.18%) | <0.001 | 1555 (25.31%) | 202 (18.76%) | <0.001 |
| College degree | 3406 (61.96%) | 1080 (62.61%) |  | 3868 (61.96%) | 618 (63.13%) |  | 3820 (62.16%) | 666 (61.84%) |  |
| High school degree | 573 (10.42%) | 248 (14.38%) |  | 667 (10.68%) | 154 (15.73%) |  | 642 (10.45%) | 179 (16.62%) |  |
| Middle school degree or below | 120 (2.18%) | 38 (2.20%) |  | 129 (2.07%) | 29 (2.96%) |  | 128 (2.08%) | 30 (2.79%) |  |
| Maternal education |  |  |  |  |  |  |  |  |  |
| Graduate degree or above | 1222 (22.23%) | 303 (17.57%) | <0.001 | 1364 (21.85%) | 161 (16.45%) | <0.001 | 1346 (21.90%) | 179 (16.62%) | <0.001 |
| College degree | 3763 (68.46%) | 1198 (69.45%) |  | 4286 (68.65%) | 675 (68.95%) |  | 4224 (68.74%) | 737 (68.43%) |  |
| High school degree | 406 (7.39%) | 192 (11.13%) |  | 477 (7.64%) | 121 (12.36%) |  | 456 (7.42%) | 142 (13.18%) |  |
| Middle school degree or below | 106 (1.93%) | 32 (1.86%) |  | 116 (1.86%) | 22 (2.25%) |  | 119 (1.94%) | 19 (1.76%) |  |
| Family income (RMB per year) |  |  |  |  |  |  |  |  |  |
| >500000 | 911 (16.57%) | 236 (13.68%) | <0.001 | 1016 (16.27%) | 131 (13.38%) | <0.001 | 1007 (16.39%) | 140 (13.00%) | <0.001 |
| 200000–500000 | 2219 (40.37%) | 617 (35.77%) |  | 2502 (40.08%) | 334 (34.12%) |  | 2469 (40.18%) | 367 (34.08%) |  |
| 100000–200000 | 1773 (32.25%) | 651 (37.74%) |  | 2044 (32.74%) | 380 (38.82%) |  | 2006 (32.64%) | 418 (38.81%) |  |
| <100000 | 594 (10.81%) | 221 (12.81%) |  | 681 (10.91%) | 134 (13.69%) |  | 663 (10.79%) | 152 (14.11%) |  |
| Gestational diabetes mellitus | 607 (11.04%) | 192 (11.13%) | 0.919 | 688 (11.02%) | 111 (11.34%) | 0.768 | 673 (10.95%) | 126 (11.70%) | 0.471 |
| Gestational hypertension | 162 (2.95%) | 72 (4.17%) | 0.012 | 196 (3.14%) | 38 (3.88%) | 0.223 | 189 (3.08%) | 45 (4.18%) | 0.059 |
| Maternal pre–pregnancy smoking | 101 (1.84%) | 46 (2.67%) | 0.033 | 119 (1.91%) | 28 (2.86%) | 0.049 | 115 (1.87%) | 32 (2.97%) | 0.018 |
| Maternal pregnancy smoking | 13 (0.24%) | 6 (0.35%) | 0.431 | 17 (0.27%) | 2 (0.20%) | 0.699 | 15 (0.24%) | 4 (0.37%) | 0.452 |
| Paternal smoking | 2197 (39.97%) | 773 (44.81%) | <0.001 | 2511 (40.22%) | 459 (46.88%) | <0.001 | 2472 (40.23%) | 498 (46.24%) | <0.001 |
| Delivery mode: cesarean | 2168 (39.44%) | 780 (45.22%) | <0.001 | 2503 (40.09%) | 445 (45.45%) | 0.002 | 2473 (40.24%) | 475 (44.10%) | 0.017 |

Abbreviations: BMI, body mass index; WHO, World Health Organization; IOTF, International Obesity Task Force.

Data are expressed as median (interquartile range) or count (percent). P value was calculated by the t-test or the rank-sum test or the Chi-squared test where appropriate.
